# Infiltration of M2 Tumor-Associated Macrophages in Oral Squamous Cell Carcinoma Correlates with Tumor Malignancy

**DOI:** 10.3390/cancers3043726

**Published:** 2011-09-28

**Authors:** Kazumasa Mori, Miki Hiroi, Jun Shimada, Yoshihiro Ohmori

**Affiliations:** 1 Division of Oral and Maxillofacial Surgery, Department of Diagnosis and Therapeutics, Meikai University of School of Dentistry, 1-1 Keyakidai, Sakado, Saitama 350-0283, Japan; E-Mails: kazu-mori@dent.meikai.ac.jp (K.M.); jsmdoms1@dent.meikai.ac.jp (J.S.); 2 Division of Microbiology and Immunology, Department of Oral Biology and Tissue Engineering, Meikai University School of Dentistry, 1-1 Keyakidai, Sakado, Saitama 350-0283, Japan; E-Mail: mikih@dent.meikai.ac.jp

**Keywords:** tumor-associated macrophage, M2 macrophage, alternatively activated macrophages, oral squamous cell carcinoma, immunohistochemical analysis, CD163, tumor microenvironment

## Abstract

Tumor-associated macrophages (TAMs) are a major cellular component in the tumor microenvironment of many solid tumors. The functional competence of TAMs varies depending on the type of tumors and their respective microenvironments. The classically activated M1 macrophages exhibit antitumor functions, whereas the alternatively activated M2 macrophages exhibit protumor functions that contribute to tumor development and progression. Although TAMs have been detected in oral squamous cell carcinoma (OSCC), little is known about their phenotype. In the present study, we performed an immunohistochemical analysis to identify TAMs in surgically resected specimens from 50 patients with OSCC and evaluated the relationship between infiltrated TAMs and the pathological grade of OSCC. Positive staining for CD163, which has been used as a marker for M2 macrophages, was observed in OSCC specimens, and the percentages of CD163^+^ cells were significantly increased based on the pathological grade. CD163^+^ cells were detected in the tumor stroma in grade I tumors, whereas an increase in the CD163^+^ cells in the tumor nest was observed in higher grades of tumors. Although infiltrated CD4^+^ and CD8^+^ T cells were detected in all pathological grades of OSCC, no correlation between the infiltrated T cells and the CD163^+^ TAMs was observed. These results indicate that the infiltrated TAMs in OSCC have an M2 phenotype and that the M2 macrophages may participate in the development of OSCC.

## 1. Introduction

Oral squamous cell carcinoma (OSCC) accounts for approximately 2% of total new cancer cases, and worldwide an estimated 128,000 people died from the disease in 2008 [1]. The incidence rate of OSCC is more than two times higher in men than in women. Epidemiological studies indicate that tobacco smoking and alcohol drinking are major risk factors for OSCC [2]. In addition to the traditional risk factors, human papilloma virus has been considered an independent risk factor for a subset of OSCCs [3].

Macrophages are involved in various aspects of host defense mechanisms and pathophysiological conditions, such as chronic inflammatory disease and cancer [4]. The functional competence of macrophages is acquired after the exposure of macrophages to stimuli in the tissue microenvironment [5]. Bacterial cellular components, such as lipopolysaccharide (LPS), and the type 1 helper T cell (Th1)-derived cytokine interferon-gamma (IFN-γ) polarize classically activated macrophages, which are referred to as M1 macrophages. These macrophages produce large amounts of proinflammatory cytokines, such as IL-12 and tumor necrosis factor-alpha (TNF-α), reactive oxygen intermediates and reactive nitrogen intermediates, which contribute to the antimicrobial and antitumor activities of macrophages [6,7]. In contrast, Th2-derived IL-4 and IL-13 induce macrophage polarization to the alternatively activated (M2) phenotype that participates in anti-inflammatory processes, tissue remodeling, scavenging effects, and angiogenesis [6,7]. These macrophages generally show low amounts of IL-12 production, impaired nitric oxide induction, enhanced expression of angiogenic cytokines, such as vascular endothelial cell growth factor (VEGF), and proteolytic enzymes. Recent studies have revealed that tumor-associated macrophages (TAMs) that are involved in tumor progression resemble the phenotype of the M2 macrophages, which contribute to tumor angiogenesis, invasion, and metastasis in various types of tumors [6]. An immunohistochemical analysis of glioma specimens has shown that an increased number of macrophages with positive staining for CD163, which is a marker for M2 macrophages in humans [6,8], correlates with the histological grade of gliomas [9]. A higher number of infiltrating CD163-positive M2 macrophages were detected in the metastasized lesion of gastrointestinal stromal tumors [10]. However, the phenotype of TAMs varies, depending on the tumor type, stage, and microenvironment. Macrophages that infiltrate tumor islets are associated with increased survival in patients with gastric cancer [11] and non-small-cell lung cancer [12,13]. Furthermore, it has been suggested that M1 macrophage densities in tumor islets may predict the survival time of patients [14,15]. Therefore, it is important to determine the phenotype of TAMs in each type of tumor to understand the process of tumor progression and to evaluate the prognosis of the patients.

Although an increased number of TAMs has been demonstrated during the progression of OSCC [16] and is associated with angiogenesis and higher histopathological grades in oral cancer [17], whether the TAMs in OSCC are the alternatively activated M2 macrophages requires further investigation. In the present study, we evaluated the status of tumor-infiltrating immune cells in tumor specimens from 50 OSCC patients. The results demonstrate that the TAMs in OSCC are CD163-positive M2 macrophages. Our results also indicate that an increased number of M2 macrophages correlate with the histopathological grade of OSCC.

## 2. Results

### 2.1. Patient Characteristics

The clinicopathological characteristics of all of the patients are summarized in Table 1. Their median age at the time of diagnosis was 55 years (range, 35–80 years), and 58% of the patients were males. The most prevalent tumor lesion was on the tongue, which comprised 58% of the OSCCs, followed by the cheek (18%), oral cavity (10%), gingiva (8%), and lip (6%). The histopathological analysis of the specimens revealed that 10 patients (20%) had grade I tumors, 21 patients (42%) had grade I to II tumors, 15 patients (30%) had grade II tumors, three patients (6%) had grade II to III tumors and one patient (3%) had grade III tumors. None of the patients had been treated with chemotherapy at the time of the diagnosis.

### 2.2. Immunohistochemical Analysis

**Figure 1.**
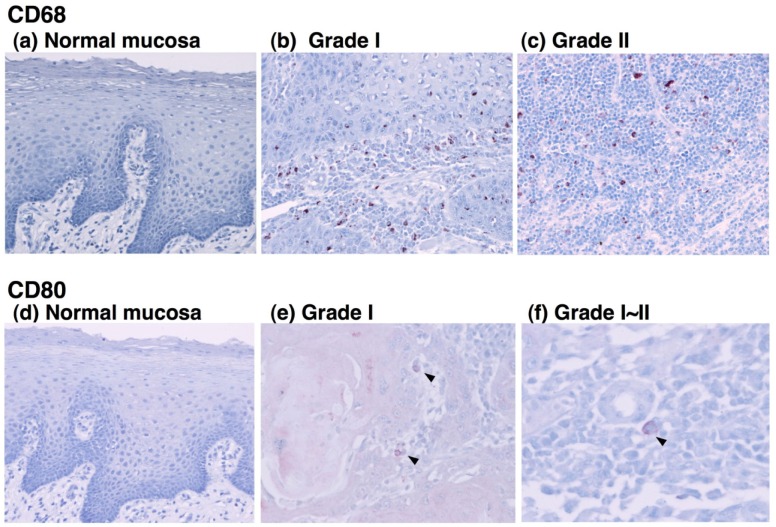
Immunohistochemical staining of OSCC with anti-CD68 and anti-CD80 antibodies. Immunoreactivity for anti-CD68 (**a-c**) and anti-CD80 (**d-f**) antibodies in normal oral mucosa (**a**, **d**; original magnification: 100×) and different pathological grades of OSCC (**b**, **c**, **e**, original magnification: 200; f: 400×) are shown. No positive cells were observed in normal mucosa (**a**, **d**). CD68^+^ macrophages were distributed in the tumoral stroma and parenchyma even in the lower-grade tumors (**b**). CD80^+^ cells were sparsely distributed in the tumoral stroma (arrow heads; **e**, **f**).

**Figure 2.**
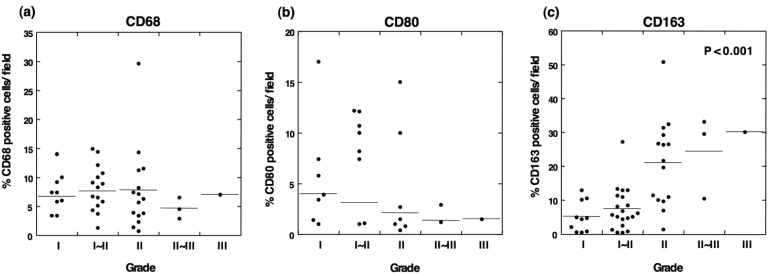
Analysis of infiltrating CD68-, CD80-, and CD163-positive cells in different histological grades of OSCC. Although CD68^+^ macrophages were observed in all grades of OSCC, no statistically significant differences were observed among the different tumor grades (**a**). Although the number of CD80^+^ M1 macrophages tended to be higher in the lower-grade tumors, no statistically significant difference was obtained (**b**). CD163^+^ M2 macrophages positively correlated with the tumor grade (**c**; p < 0.001, Kruskal-Wallis test).

To determine the type of infiltrated immune cells in OSCC, we performed an immunohistochemical analysis using antibodies against lineage-specific markers such as CD68 for all macrophages [18], CD80 for M1 macrophages [6,19], CD163 for M2 macrophages, CD4 for helper T lymphocytes, and CD8 for cytotoxic T lymphocytes. The CD80 antigen, which is known as B7-1, is a costimulatory molecule that expresses activated macrophages and dendritic cells and has been used as an M1 macrophage marker [6,19-21]. CD163 is a scavenger receptor for hemoglobin and is exclusively expressed in the monocyte-macrophage lineage cells [8]. In particular, a high expression of CD163 is observed in alternatively activated M2 macrophages that participate in the regulation of anti-inflammatory responses [22,23]. In the current study, the immunohistochemical analysis demonstrated that although no positive staining for CD68 was detected in normal oral mucosa, CD68^+^ cells were observed in all grades of tumor specimens (Figures 1 and 2a). The CD68^+^ macrophages were distributed in the tumor stroma and parenchyma, even in the grade I OSCC (Figure 1b). However, no correlation was observed between the number of infiltrating CD68^+^ cells and the histological grade of the tumor (Figure 2a).

**Figure 3.**
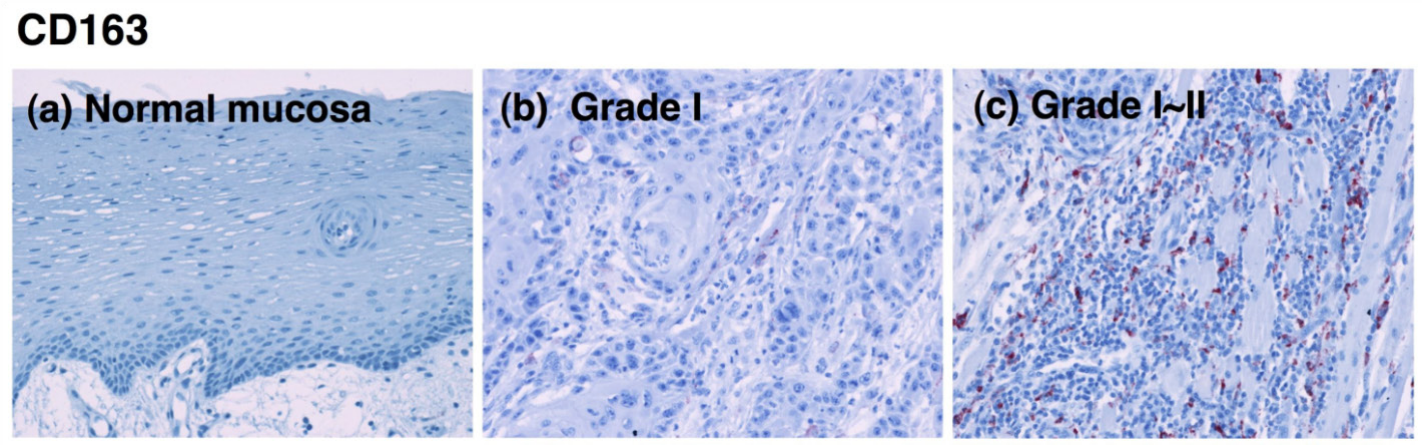

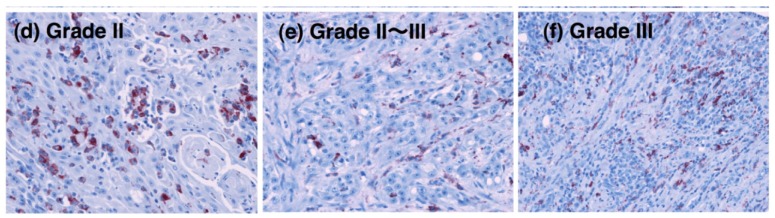
Immunohistochemical staining of OSCC with the anti-CD163 antibody. Immunoreactivity for the anti-CD163 antibody in normal oral mucosa (**a**, original magnification: 100×) and different pathological grades of OSCC (**b-f**, original magnification: 200×) are shown. No positive cells were observed in normal mucosa (**a**); CD163^+^ cells were mainly observed in the tumor stroma in grade I OSCC (**b**); and the distribution of CD163^+^ cells increased in the tumor nest with increasing OSCC grade (**d-f**).

CD80-positive M1 macrophages were rarely detected in specimens from the lower-grade OSCCs (Figure 1e). Although the proportion of CD80^+^ cells decreased in specimens from higher grades of the tumor [Figure 2(b)], no statistical significance was obtained. CD163-positive M2 macrophages were also detected in specimens from the lower-grade OSCCs (Figures 3b and 3c). However, the number of infiltrated M2 macrophages was significantly increased in specimens from higher grades of OSCC (Figures 3d-3f). The CD163^+^ M2 macrophages were mainly distributed throughout the tumoral stroma, but not the tumor nest, in grade I OSCC (Figure 3b).

However, the proportion of infiltrating M2 macrophages in the tumor parenchyma was significantly increased in specimens from the higher-grade OSCCs (Figures 3d-3f). Using the Kruskal-Wallis test, the statistical analyses showed that the number of infiltrating CD163-positive cells correlated with the histological grade of the malignancy (p < 0.001; Figure 2c). These results indicate that the number of CD163-positive macrophages that infiltrated the tumor parenchyma correlates with the grade of tumor malignancy in OSCC.

**Figure 4.**
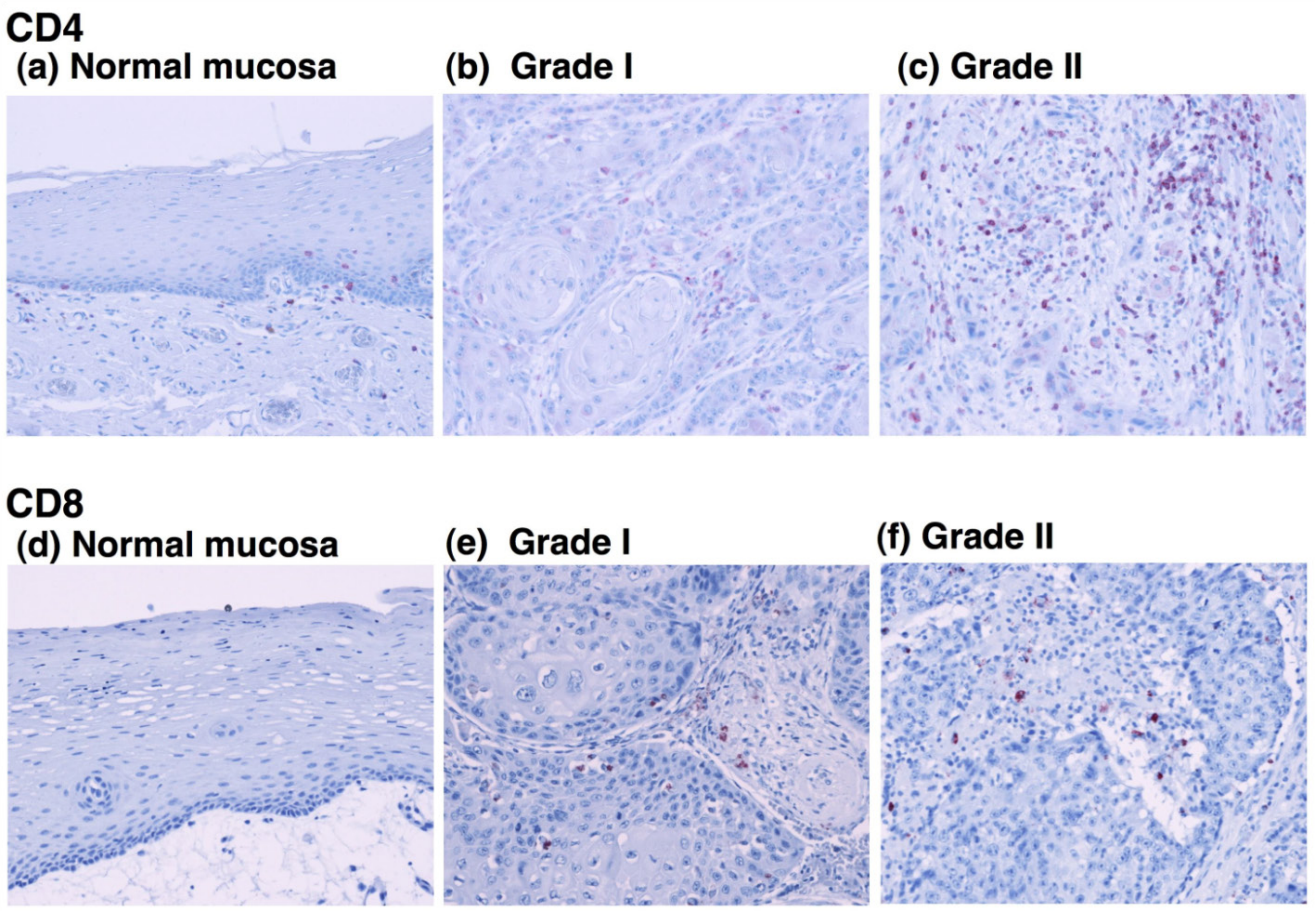
Immunohistochemical staining of OSCC with anti-CD4 and anti-CD8 antibodies. Immunoreactivity for anti-CD4 (**a-c**) and anti-CD8 (**d-f**) antibodies in normal oral mucosa (**a**, **d**, original magnification: 100×) and different pathological grades of OSCC (**b**, **c**, **e**, **f**, original magnification: 200×) are shown. Many CD4^+^ T cells were detected in the tumor stroma in all stages of OSCC.

To determine the relationship between the infiltration of M2 macrophages and T lymphocytes, we evaluated the infiltration ability of CD4^+^ and CD8^+^ T cells in OSCC. Immunohistochemical staining showed that CD4-positive T cells were detected in all grades of OSCC and that the CD4^+^ cells were predominantly observed in tumor stroma (Figures 4b and 4c).

**Figure 5.**
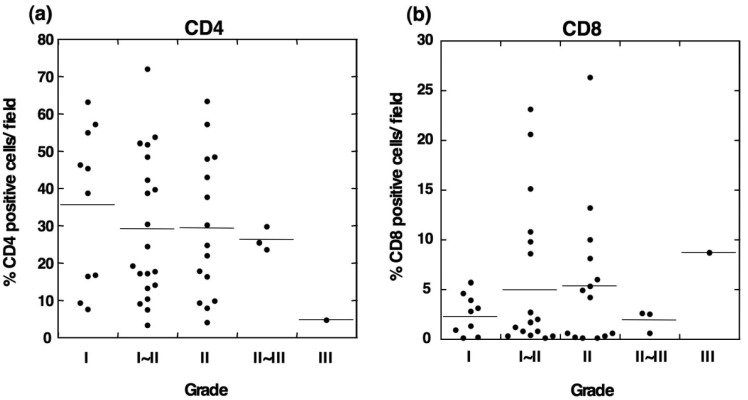
Analysis of infiltrating CD4- and CD8-positive cells in different histological grades of OSCC. Although CD4^+^ (**a**) and CD8^+^ (**b**) T cells were observed in OSCC, no statistically significant differences were observed among the different tumor grades.

Statistical analysis showed no correlation between the number of CD4^+^ cells and the tumor grade (Figure 5a). Although CD8-positive T cells were also observed in OSCC (Figures 4e and 4f), the number of infiltrated CD8^+^ cells was smaller than that the number of CD4^+^ cells. The densities of CD8^+^ cells varied among the patients, and no statistical differences were observed between the number of infiltrated CD8^+^ cells and the tumor grade (Figure 5b). These results suggest that the T cells in OSCC do not directly correlate with the number of infiltrating M2 macrophages.

## 3. Discussion

The tumor-associated macrophage is a predominant cellular component for the tumor microenvironment in various tumors. The M2 macrophages are TAMs that are associated with protumor activities, including the development and progression of solid tumors. Although previous studies have demonstrated that TAMs are detected in OSCC [16,17], whether these TAMs have the M1 or M2 phenotype is poorly understood. In the present study, we evaluated surgically dissected specimens from 50 OSCCs using an immunohistochemical analysis with the anti-CD80 antibody, which is a marker for M1 macrophages, and the anti-CD163 antibody, which is a marker for M2 macrophages [6,8]. The results demonstrate that CD80^+^ M1 macrophages are rarely observed in OSCC, whereas many CD163^+^ M2 macrophages are detected in OSCC. In addition, the results reveal that the densities of infiltrating CD163^+^ cells increase with the pathological grade of the tumor. To the best of our knowledge, the present study is the first report to identify the M2 macrophage in OSCC and to evaluate the relationship between infiltrating M2 macrophages and the tumor grade in OSCC.

The TAMs with the M2 phenotype are involved in tumor angiogenesis, invasion and metastasis in many tumors [24]. The increased number of TAMs correlates with VEGF expression and the microvessel density in breast cancer [25]. TAMs also exhibit an immunosuppressive phenotype by producing a large amount of IL-10, which dampens the expression of IL-12 [26]. A recent study has demonstrated that M2 macrophages induce CD4^+^ regulatory T cells (Treg) to mediate immunosuppression [27]. Although the precise role of the M2 macrophages in OSCC is unknown, these lines of evidence suggest that the M2 macrophages in OSCC have protumor activities that participate in cancer development and progression.

In the present study, we observed that the number of M2 macrophages increased in cases with higher grades of OSCC. Although the exact reason for the increased M2 macrophages in higher-grade OSCCs is currently unknown, we propose several potential mechanisms. First, the higher grade of the tumor and its tumor microenvironment may facilitate differentiation of TAMs into the M2 phenotype by producing cytokines and chemokines [26,28-31]. In this regard, activated macrophages were originally induced *in vitro* using Th2-derived IL-4 [32], and later studies have shown that anti-inflammatory cytokines and agents, such as IL-13, IL-10 or glucocorticoid, also induce M2 macrophages [22,33]. IL-10 and VEGF have been detected in head and neck squamous cell carcinomas, and a higher expression of these cytokines correlates with the tumor grade and progression as well as a reduced patient survival time [34-36]. Furthermore, CCL2 (known as monocyte chemoattractant protein-1) and IL-6 promote the survival of human monocytes and induce M2-macrophage differentiation [37]. Many cancer types, including oral squamous cell carcinoma, exhibit constitutive expression of CCL2 and IL-6 [38,39], whereas normal oral mucosal epithelial cells express lower levels of these cytokines and chemokines [40,41]. Therefore, higher tumor grades and the tumor microenvironment may exhibit increased levels of various cytokines and chemokines that promote monocyte/macrophage survival and differentiation into the M2 phenotype.

Although the CD68-positive cells indicated the total population of macrophages in the OSCC specimens, there was no correlation between the number of CD68^+^ cells and the tumor grade. Because CD68^+^ cells include M1, M2 and undifferentiated monocytes/macrophages, it is likely that these mixed cell populations are functionally heterogeneous regarding the development and progression of OSCC. In addition, CD80^+^ M1 and CD163^+^ M2 macrophages were detected in the same specimens from patients with lower grades of OSCC. Although no statistically significant differences were observed, the CD80^+^ cells tended to decrease with the tumor grade (Figure 2b). We observed a weak negative correlation between the number of CD80^+^ and CD163^+^ cells (r = 0.2965, p = 0.0688, Spearman's rank coefficient, data not shown). Whether the increase in the CD163^+^ M2 macrophages is partially derived from dedifferentiating CD80^+^ M1 macrophages during the development of the higher-grade tumors is unknown.

Previous studies have demonstrated that CD4^+^ T has a regulatory role in M2 macrophage development [32,42,43]. In a mouse mammary tumor model, IL-4-expressing CD4^+^ T cells induced TAMs with the M2 phenotype to enhance tumor invasion and metastasis [42]. In addition, CD4^+^CD25^+^Foxp3^+^ regulatory T cells induce the alternative activation of human monocytes or macrophages [43]. To determine the relationship between TAMs and infiltrating T cells in OSCC, we assessed the distribution of CD4^+^ T cells using an immunohistochemical analysis. Although the infiltration of CD4^+^ cells was observed in OSCC, there was no statistically significant difference between the numbers of infiltrating CD4^+^ T cells and CD 163^+^ macrophages. These results suggest that the infiltrating CD4^+^ T cells are not directly involved in the polarization of TAMs in OSCC. Because tumor cells produce various anti-inflammatory cytokines and growth factors, such as IL-6, IL-10, IL-13, VEGF, and M-CSF [26,31], OSCC-derived immunosuppressive cytokines may modulate infiltrating TAMs, leading to the M2 phenotype. Further studies are required to determine the functional role of tumor-derived cytokines in the polarization of TAMs in OSCC.

CD8^+^ cytotoxic T cells play a crucial role in antitumor immunity in various types of tumors. An increase in the CD8^+^ cells in the tumor nest correlates with improved patient survival or certain types of tumors [44,45]. However, previous studies have demonstrated that increased numbers of intratumoral CD8^+^ cells correlate with the tumor grade of renal cell carcinomas [46]. In the present study, we detected a number of infiltrating CD8^+^ T cells in OSCC However, the number of infiltrating CD8^+^ cells did not correlate with the tumor grade of OSCC or with the number of infiltrating M2 macrophages. The infiltration of CD8^+^ T cells in the tumor nest is not necessarily functionally active [47]. Previous studies have shown that the tumor-infiltrating T cells are functionally inactivated in certain types of tumors [48,49]. In the early stages of tongue cancer, intraepithelial CD8^+^ T cells and NK cells display predominantly immunosuppressed phenotypes [50]. Similar to the effects on M2 macrophages, tumor cell-derived cytokines and mediators, such as IL-10, TGF-β, and PGE2, have immunosuppressive effects on the infiltrating lymphocytes [47,51]. Therefore, these lines of evidence suggest that the tumor microenvironment in OSCC may create an immunosuppressive microenvironment and that the M2 phenotype of TAMs may contribute to further development of OSCC.

## 4. Materials and Methods

### 4.1. Tissue Samples

Specimens were surgically obtained from 50 patients with OSCC and 10 patients with normal oral epithelium at the Department of Oral and Maxillofacial Surgery at the Meikai University School of Dentistry. The current study was performed according to the guidelines of the Ethics Committee of Meikai University. These tissues were fixed for 24–48 h in 4% formaldehyde that was freshly prepared from paraformaldehyde in phosphate-buffered saline (PBS) at 4 °C. Tissue specimens were sliced into 4-μm sections and mounted onto 3-aminopropyltriethoxysilane–coated glass slides. Hematoxylin and eosin slides were examined, and the histological diagnosis was reevaluated based on the WHO Classification of Head and Neck Tumors [52,53]. Grade I is a well-differentiated phenotype with the histological features that closely resemble those of the squamous epithelial lining of the oral mucosa. In addition, other features include: varying proportions of basal and squamous cells with intercellular bridges; keratinization and the epithelial pearls; rare instances of mitotic (normal or atypical) or multinucleated epithelial cells and minimal nuclear and cellular pleomorphism. Grade II is an intermediate differentiated phenotype. Keratinization and intercellular bridges are less conspicuous. The epithelial pearls are rarely observed, whereas nuclear and cellular pleomorphism are more prominent. Grade III is a poorly differentiated phenotype with histological features that slightly resemble those of the normal stratified squamous epithelium of the oral mucosa. Keratinization and intercellular bridges are observed. Atypical mitoses, cellular and nuclear pleomorphism as well as many multinucleated cells are detected.

### 4.2. Immunohistochemical Staining

The tissue sections were deparaffinized and immersed in 0.01 M citrate buffer (pH 6.0) and heated in a microwave oven for 15 min for antigen retrieval. After rinsing in PBS, the sections were incubated with 3% hydrogen peroxide in methanol for 10 min to block endogenous peroxidase activity. Endogenous avidin and biotin were blocked using the Avidin/Biotin Blocking Kit (Zymed Laboratories, San Francisco, CA, USA) at room temperature. To reduce nonspecific antibody binding, the samples were exposed to 20% bovine serum albumin for 60 min. The primary antibodies mouse monoclonal anti-human CD68 (clone PG-M1, Dako, Kyoto, Japan), anti-human CD80 (clone 37711, R&D Systems, Minneapolis, MN, USA), anti-human CD163 (clone 10D6; Leica Microsystems, Wetzlar, Germany), rabbit monoclonal anti-human CD4 (clone SP35; Cell Marque, Austin, TX, USA) or mouse monoclonal anti-human CD8 (clone C8/114B; Dako) antibodies were used at a dilution of 1:100 in a humidified chamber overnight at 4 °C. After incubation with the primary antibody, tissue sections were washed in PBS and incubated with horseradish peroxidase-labeled anti-mouse or anti-rabbit antibodies (Dako EnVision System, HRP-Labeled Polymer, Dako, Kyoto, Japan) for 30 min. The peroxidase activity was visualized by immersing the tissue sections using the AEC Substrate Kit (Dako), which produced a brown reaction product. Finally, the tissue sections were counterstained with Mayer's hematoxylin and mounted. For a negative control, mouse IgG (Sigma-Aldrich Co., St. Louis, MO, USA) was used in place of the primary antibody.

To evaluate positively stained cells after incubation with each antibody, three high-power magnification fields (400×) with the most abundant distribution of positive cells were selected from each specimen. The positively stained and unstained cells were counted. The data were expressed as the mean percentage of the ratio of the number of positive cells relative to the total number of cells for one microscopic field (400×).

### 4.3. Statistical Analysis

The statistical differences of positively stained cells among the different tumor grades were tested using the Kruskal-Wallis nonparametric test.

## 5. Conclusions

The infiltrated TAMs in OSCC exhibited the M2 phenotype, and the alternatively activated M2 macrophages were significantly increased with the increasing pathological grade of the tumor. The infiltration of the M2 macrophages may participate in the development and progression of OSCC.

## Figures and Tables

**Table 1.** Clinicopathological characteristics of the patients with OSCC.

		**Number of patients (%)**
Age	Median (35–80)	55.0
	50 y <	11 (22)
	50 y >	39 (78)
Gender	Female	21 (42)
	Male	29 (58)
Grade	I	10 (20)
	I∼II	21 (42)
	II	15 (30)
	II∼III	3 (6)
	III	1 (2)
Region	Tongue	29 (58)
	Cheek	9 (18)
	Oral cavity	5 (10)
	Gingiva	4 (8)
	Lip	3 (6)
